# GIT1 regulates synaptic structural plasticity underlying learning

**DOI:** 10.1371/journal.pone.0194350

**Published:** 2018-03-19

**Authors:** Amanda C. Martyn, Krisztian Toth, Robert Schmalzigaug, Nathan G. Hedrick, Ramona M. Rodriguiz, Ryohei Yasuda, William C. Wetsel, Richard T. Premont

**Affiliations:** 1 Department of Medicine, Duke University Medical Center, Durham, North Carolina, United States of America; 2 Department of Neurobiology, Duke University Medical Center, Durham, North Carolina, United States of America; 3 Department of Psychiatry and Behavioral Sciences, Duke University Medical Center, Durham, North Carolina, United States of America; 4 Mouse Behavioral and Neuroendocrine Analysis Core Facility, Duke University Medical Center, Durham, North Carolina, United States of America; San Raffaele University and San Raffaele Scientific Institute, ITALY

## Abstract

The signaling scaffold protein GIT1 is expressed widely throughout the brain, but its function *in vivo* remains elusive. Mice lacking GIT1 have been proposed as a model for attention deficit-hyperactivity disorder, due to alterations in basal locomotor activity as well as paradoxical locomotor suppression by the psychostimulant amphetamine. Since we had previously shown that GIT1-knockout mice have normal locomotor activity, here we examined GIT1-deficient mice for ADHD-like behavior in more detail, and find neither hyperactivity nor amphetamine-induced locomotor suppression. Instead, GIT1-deficient mice exhibit profound learning and memory defects and reduced synaptic structural plasticity, consistent with an intellectual disability phenotype. We conclude that loss of GIT1 alone is insufficient to drive a robust ADHD phenotype in distinct strains of mice. In contrast, multiple learning and memory defects have been observed here and in other studies using distinct GIT1-knockout lines, consistent with a predominant intellectual disability phenotype related to altered synaptic structural plasticity.

## Introduction

The GRK-interacting (GIT) proteins, GIT1 and GIT2, are signaling adaptor proteins that also function as GTPase-activating proteins (GAPs) for the ADP-ribosylation factor (Arf) small GTP-binding proteins [1, 2]. GIT proteins bind tightly with p21-activated kinase [PAK]-interacting exchange factor (PIX) proteins to form oligomeric GIT/PIX signaling scaffold complexes [3–6]. The two PIX proteins, α-PIX and β-PIX, are Rho family guanine nucleotide exchange factors (GEFs) that activate the p21 Rac1/Cdc42 small GTP-binding proteins and scaffold the conventional p21-activated kinases (PAKs 1–3) [7]. The GIT/PIX complex is recruited to specific cellular locations in response to extracellular signals, including to both pre-synaptic and post-synaptic neuronal membranes [8–12]. At these locations, GIT/PIX complexes function to regulate Arf and Rac1/Cdc42 signaling, and as scaffolds for a large number of signaling partners, but importantly including PAKs [2].

GIT proteins are widely expressed throughout the brain [13], but to date, relatively little is known of the role of GIT proteins in complex signaling events in neurons. Based on interactions with α-PIX and PAK3, known human X-linked intellectual disability genes [14, 15], GIT1 was suggested to regulate cognitive function through regulation of synaptic plasticity, and experiments in primary neurons have indicated synaptic roles [8, 16]. We previously reported that GIT1 knockout mice exhibit numerous normal behaviors related to anxiety, depression and gross locomotor function, but exhibit very poor memory performance after fear conditioning [17]. Others have reported very poor performance by a distinct GIT1-knockout line in operant conditioning to a taste reward [18], consistent with poor learning ability. However, a genetrap mouse strain lacking GIT1 was reported to model human ADHD, in addition to exhibiting learning deficits [19]. In particular, loss of GIT1 was associated with two critical aspects of ADHD. First, GIT1-genetrap mice were reported to exhibit basal hyperactivity. Second, GIT1-genetrap mice were reported to respond to amphetamine or methylphenidate with locomotor suppression rather than locomotor activation, consistent with effects of these psychostimulant drugs to calm and focus ADHD patients. However, locomotor hyperactivity and psychostimulant-induced locomotor suppression were only evident during the night phase of the diurnal cycle and only in young adult mice. This is in contrast to our earlier report that GIT1-knockout (KO) mice exhibit no hyperactivity in the open field [17], when tested as older adults during the day.

Here we set out to assess hyperactivity in young individuals from our GIT1-KO mouse line. We show that neither critical aspect of ADHD-like behavior, basal hyperactivity or amphetamine-induced locomotor activity depression, is apparent in our line of GIT1-KO mice. GIT1-KO mice do, however, display learning and memory deficits in both short- and long-term memory testing. Loss of GIT1 leads to reduced density and structural plasticity of hippocampal synapses, which may contribute to the poor hippocampus-regulated cognitive function observed in these mice.

## Materials and methods

### Animals

GIT1-knockout and littermate wildtype and heterozygote mice used in this study were 2- to 4-months of age, derived from breeding heterozygotes on a mixed c57 x 129 background, as previously described [20]. One set of studies was performed on a distinct genetic background derived as follows: mixed strain *Git1* heterozygote mice were bred with c57Bl/6J mice (Jackson Labs, Bar Harbor, Maine, USA) for >10 generations, and these c57-backcrossed *Git1* heterozygotes were bred with 129P2/OlaHsd mice (Harlan Envigo, Indianapolis, Indiana, USA) to generate F1 c57 x Ola heterozygotes that were interbred to generate F2 c57 x Ola knockout and littermate wildtype mice for testing. This F2 c57 x Ola genetic background was chosen to match that used by Won et al [19] in an attempt to assess the role of genetic background effects on ADHD-like behavior in the absence of GIT1. 129P2/OlaHsd mice have been reported to exhibit lower basal locomotion than c57Bl/6 [21]. Mice bearing the *Git1*/flox allele were generated by Cre deletion of gene-targeted founder mice as described for the *GIT1*/del allele [20], and backcrossed to the c57Bl/6J background for >10 generations prior to use. Mice were group-housed in temperature- (22°C) and humidity- (45%) controlled rooms with a 12:12 light-dark cycle (lights on at 0700 h) with food and water available *ad libitum*. Behavioral experiments were performed between 0900 and 1600 hours in the light cycle, with the exception of the 24-hr locomotor activity tests and dark-phase amphetamine test (at 2000 to 2200 hours). Male and female mice were used in all studies. While GIT1 deficiency leads to perinatal lethality in our strain used here [20] and in two distinct GIT1-KO alleles [18, 22], we observe no additional mortality in adult mice up to 1 year of age. All animals were treated in accordance with NIH Guidelines for the Care and Use of Animals following an animal protocol approved by the Duke University Institutional Animal Care and Use Committee.

### Locomotion

Mice were acclimated to the testing room in the home cage for 1 hour prior to beginning the test, and had not experienced a cage change for at least 24 hours. Mice were placed in the center of a 20 cm x 20 cm arena with 30 cm high walls (AccuScan Instrument, Inc., Columbus, OH, USA) and allowed to freely explore the arena. Distance traveled was used as a measure of locomotion, and activity was automatically recorded by the Versamax software (AccuScan Instruments). Diurnal locomotor activity was measured over 30 min intervals and quantified using cumulative counts in light or dark periods. Pharmacological assessment of locomotion was measured over 5 min intervals and using cumulative counts for the 180 minutes following drug administration. A two-way repeated measure ANOVA and Tukey’s Multiple Comparison test was used to test for statistical significance. In the pharmacological tests, 2 mg/kg or 4 mg/kg amphetamine or saline (control) was administered by intraperitoneal (i.p.) injection 60 min after the mice had been placed in the arena.

### Rotarod performance

Balance and coordination were examined using an accelerating (4–40 rpm over 5-min) Rotarod on the first day, followed 24 hrs later with a steady speed test (20 rpm) (Med-Associates, St. Albans, VT, USA). On each test day, the mice were given 4 successive 5-min trials that were separated by 30 min. The latency to fall was scored when the mouse fell from the rod (prior to 300 sec). If the mice did not fall from the rotating rod, the latency to fall was scored as “300” seconds.

### Grip strength

Forelimb, hindlimb, and whole body grip strength were measured with a Mouse Grip Strength Meter (San Diego Instruments, San Diego, CA, USA). Briefly, the mouse is allowed to grip a small stainless steel grid, which is fitted to a peak amplifier unit that records the pull force of the animal in grams. Animals were tested in three sequential trials, separated by 15 sec (± 5 sec). A final grip strength score is calculated from the average of the 3 trials for each animal.

### Object recognition

This test was conducted over 2 days as described previously [23]. Object recognition consisted of training and a short-term memory (STM) test on day 1 and a long-term memory (LTM) test on day 2. Training and test sessions were each 5 min in duration and involved the presentation of two objects made from plastic (4 × 3 × 2 cm) placed into opposite corners of a solid-walled acrylic arena (20 × 20 × 30 cm) and affixed to the floor with double-sided tape. On the first day, mice were exposed to a pair of identical (“familiar”) objects. In the STM (20 min after training) and LTM (24 hr after) tests, one familiar object was replaced with a distinct novel object of the same dimension. All tests were videotaped, and the total time spent interacting with each of the two objects was scored by trained observers blinded to the genotypes of the animals, using Noldus Observer (Noldus Information Technologies, Leesburg, Virginia, USA). Time spent with an object included direct visual orientation towards an object while being within one-body length of that object, and sniffing, touching, or climbing on the object. Recognition scores were calculated by subtracting the total time with the familiar object from time spent with the novel object, and dividing this difference by the total amount of time spent with both objects. Positive scores indicate preference for examining the novel object, negative scores indicate preference for the familiar object, and a score of “zero” indicates no preference between novel or familiar object.

### Morris water maze

Spatial learning and memory were examined in the Morris water maze as described previously [24]. Briefly, training and testing were conducted under ~125 lux illumination in a 120 cm diameter stainless-steel pool filled with water, made opaque with white non-toxic poster paint (Crayola LLC, Easton, Pennsylvania, USA), and maintained at 24°C. The pool was divided into four quadrants; northeast (NE), northwest (NW), southeast (SE) and southwest (SW). Before testing, mice were handled for 10 min and then acclimated to standing in water for 1 min for 5 consecutive days. Next, for one day mice were trained to sit on the hidden platform (1 cm below the water’s surface and 20 cm from the rim of the pool) in the NE quadrant for 20 s and then allowed to swim freely for 60 s before being returned to the platform for 15 s. On the following day, water-maze testing commenced. To test spatial acquisition, mice were exposed to 4 trials per day in pairs that were separated by 60 min (days 1 to 6), with the hidden platform in the NE quadrant. Release points were randomized across test-trials and test-days. On days 2, 4 and 6, a single probe trial was given 1 hr after the 4 test-trials, where the platform was removed. An independent set of mice was used for visible platform testing conducted over 4 trials per day for 5 consecutive days. Here, the mice were released from the northern-most point and given 60 s to swim to the visible platform (a 5 x 5 cm patterned flag was suspended 24 cm above the platform). Except for probe trial durations that were preset for 60 s, all trials ended when the animal located the platform or after 60 s of swimming. Performance on all tests was scored by Ethovision XT7 (Noldus Information Technologies Inc., Leesburg, Virginia, USA) using video recorded by a high-resolution camera suspended 6 ft above the center of the pool. Tracking profiles were generated by Ethovision software.

### Golgi staining and spine quantification

GIT-1 WT and KO mice were deeply anaesthetized with urethane and briefly perfused through the heart with 0.9% saline. The brains were removed and placed in vials containing Golgi A+B solution (FD Rapid GolgiStain Kit, FD NeuroTechnologies, Columbia, Maryland, USA) and after 14 days transferred to vials containing Golgi C solution. After at least 3 days in Golgi C solution, the brains were rapidly frozen in isopentane pre-cooled with dry ice and cut into 200-μm thick coronal sections using a sliding microtome, and stained using procedures described in the kit. Brain slices were imaged with a widefield deconvolution microscope (DeltaVision Elite). Neuronal spine density in CA1 region of the hippocampus and in the 5th cortical layer was counted using ImageJ software.

### Structural plasticity of single dendritic spines in hippocampal CA1 neurons

Hippocampal slices (350 μm) were isolated from *Git1*^flox/flox^ mice at 5–8 days of age, cultured, and transfected with GFP plus Cre recombinase fused to tdTomato, or with GFP alone as a control, at 8–12 days in vitro using biolistic transfection, as described previously [25–27]. Individual spines of transfected CA1 neurons were visualized using 2-photon fluorescence imaging with a Ti-Sapphire laser (MaiTai, Spectra-Physics, Santa Clara, California, USA) tuned to a wavelength of 920nm. All samples were imaged using a laser power < = 2mW as measured at the objective. Fluorescence emission was collected using an immersion objective (60x, numerical aperture 0.9, Olympus), divided with a dichroic mirror (565 nm), and detected with a photoelectron multiplier tube (PMT) placed downstream of a wavelength filter (Chroma, HW510). All experiments were performed 9–12 days after transfection, based on preliminary studies indicating that the structural plasticity phenotype stabilized after 8 days. Glutamatergic stimulation of individual dendritic spines was achieved via 2-photon photolysis of 4-methoxy-7-nitroindolinyl-caged-L-Glutamate (MNI-caged glutamate) with a second Ti-Sapphire laser tuned to 720 nm. Experiments were performed in Mg^2+^-free artificial cerebrospinal fluid (ACSF; 127mM NaCl, 2.5mM KCl, 4mM CaCl_2_, 25mM NaHCO_3_, 1.25mM NaH_2_PO_4_, and 25mM D-glucose) containing 1μM tetrodotoxin (TTX) to inhibit action potentials, and 4mM MNI-caged glutamate, and aerated with a 95%O_2_/5%CO_2_ mixture at 30°C, as described previously. Individual spines were chosen to be of similar size at baseline. Spine size was quantified using ImageJ from sequential fluorescence images, using the background-subtracted integrated fluorescence intensity (*F*) over a region of interest manually drawn around the targeted spine. The change in spine volume was measured as *F/F*_*0*,_ in which *F*_*0*_ is the average fluorescence intensity prior to glutamate uncaging.

### Statistical analysis

Data were analyzed by a standard one-way or two-way ANOVA test for comparison between genotypes, treatments, or doses (GraphPad Prism 6 software). Individual genotypes, treatments, or doses were compared using post-hoc test whenever ANOVA showed significance to either genotype or genotype x time interaction. A probability value of p<0.05 was considered as statistically significant. Statistical results are described in the figure legends. All data are presented as mean ± SEM.

## Results

### GIT1-KO mice fail to model ADHD

**Fig 1:**
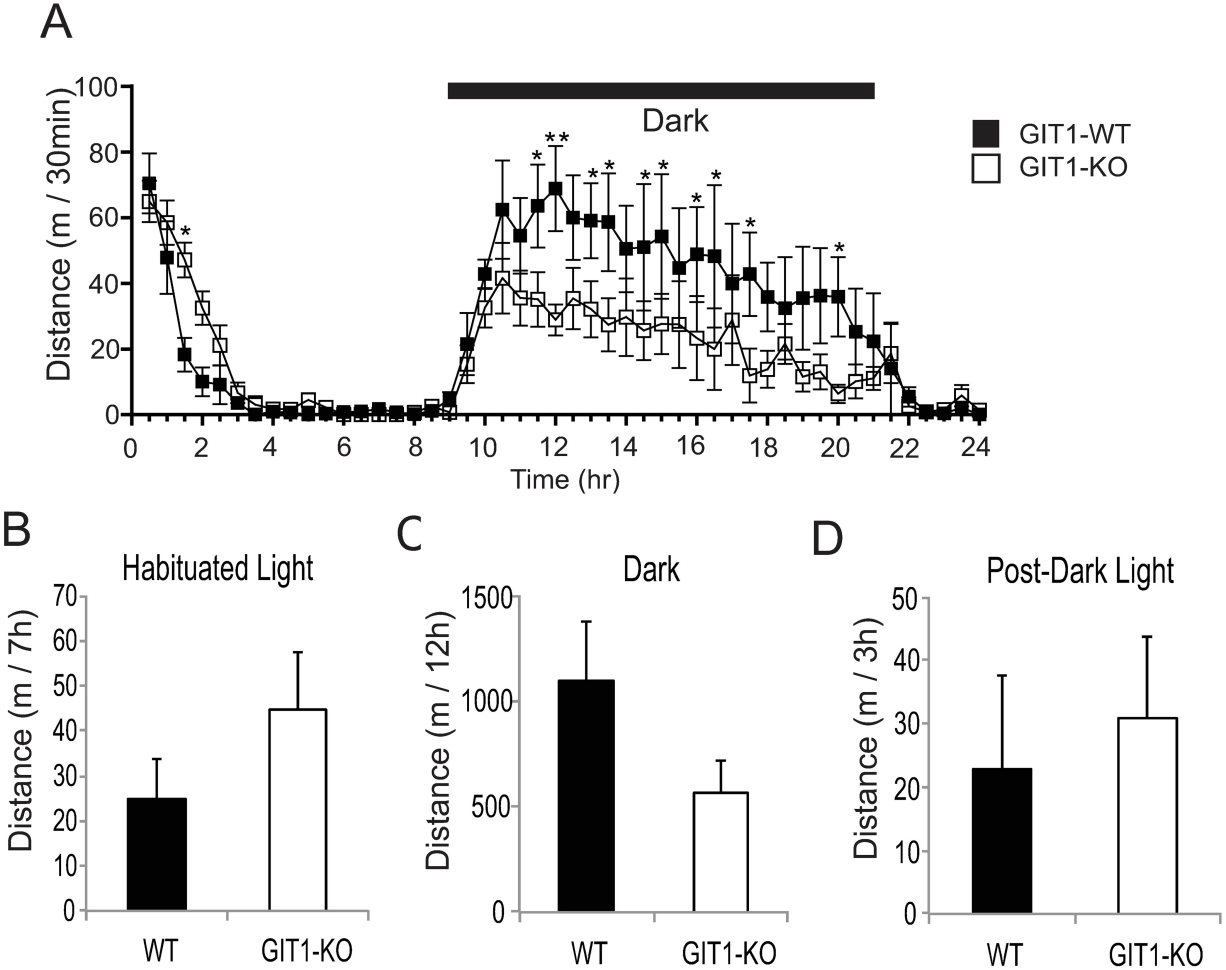
Spontaneous locomotor activity in GIT1 deficient mice. A) GIT1 WT (n = 6, black square) and KO (n = 6, open square) mice on a mixed genetic background were subjected to the open field test for 24 hours under the normal 12hr-12hr light-dark cycle. Distance is shown in 30 min windows. * p<0.05, **p<0.01 in a two-way repeated measures ANOVA within time using a Holm-Sidak post-hoc test. B-D) Total distance was summed for the light (inactive) phase prior to the dark phase, but after initial habituation for 2h (B), for the entire dark (active) phase (C), and for the light (inactive) phase following the dark phase (D). No significant differences between WT (black bars) and KO (open bars) were observed for any total distances.

To assess basal activity, we subjected GIT1 KO and WT mice on a mixed c57/129 genetic background to the open field test for 24 hours under the normal 12hr-12hr light-dark cycle. Once habituated to novelty of the test chamber, wildtype and GIT1-KO mice both display low activity in the light phase, but elevated activity in the dark phase of the diurnal cycle (Fig 1). However, rather than hyperactivity, GIT1 KO mice exhibited reduced spontaneous locomotor activity in the dark phase at some time points (Fig 1A), but when activity was measured as total distance traveled, GIT1 KO activity was statistically indistinguishable from that of wildtype littermates during the dark or the light phases (Fig 1B, 1C and 1D).

**Fig 2:**
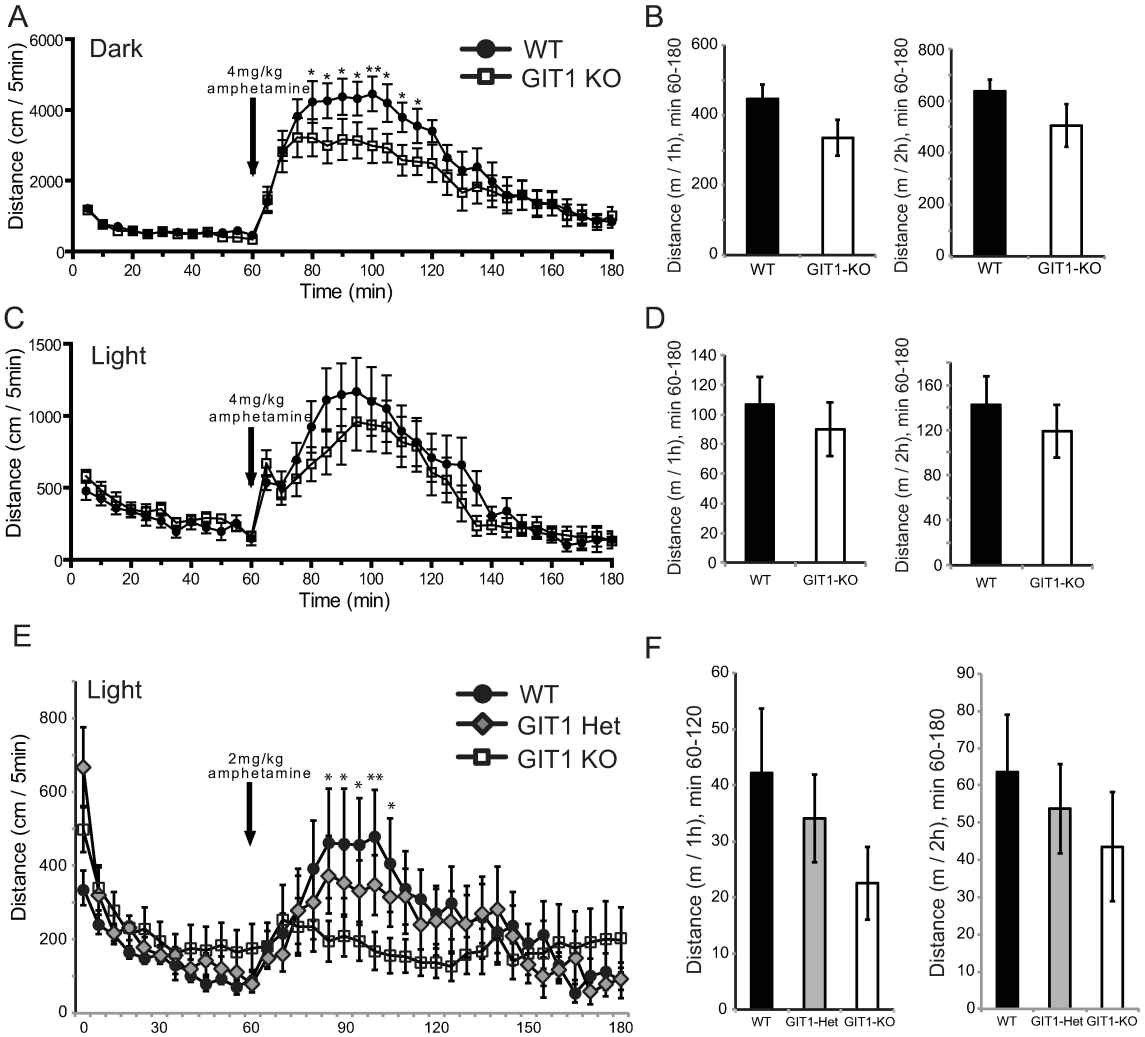
Amphetamine-induced locomotor activity in GIT1 deficient mice. GIT1 WT (black circle) and GIT1 KO (open square) mice on a mixed genetic background were habituated to the locomotor chamber for 60 min prior to amphetamine injection. A) GIT1 WT (n = 9) and GIT1 KO (n = 8) mice were injected with 4mg/kg amphetamine in their active (dark) phase, and activity is shown in 5 min windows. B) Drug-induced locomotor activity (dark phase, 4mg/kg amphetamine) was totaled over 1h and 2h following injection. No difference between WT and KO. C) GIT1 WT (n = 6) and GIT1 KO (n = 5) mice were injected with 4mg/kg amphetamine in their inactive (light) phase. D) Drug-induced locomotor activity (light phase, 4mg/kg amphetamine) was totaled over 1h and 2h following injection. No difference. E) GIT1 WT (n = 8), GIT1 KO (n = 7) and GIT1 heterozygote (Het, n = 5) mice were injected with 2mg/kg amphetamine in their inactive (light) phase. F) Drug-induced locomotor activity (light phase, 2mg/kg amphetamine) was totaled over 1h and 2h following injection. No difference. p<0.001 in a two-way repeated measures ANOVA between genotypes over time; * *p*<0.05, ** *p*<0.01, *** *p*<0.001 within time using a Holm-Sidak post-hoc test.

Next, we challenged GIT1 KO mice with amphetamine, a psychostimulant known to increase locomotion. First, we tested the effect of amphetamine administration during the dark (active) phase of the diurnal cycle, and found that a high dose of 4 mg/kg amphetamine (as used by Won *et al*., [22]) greatly increased locomotor activity of young GIT1 KO mice as well as of control wildtype mice (Fig 2A). While amphetamine produced a reduced locomotor effect in GIT1-KO mice at multiple time-points, the overall distance travelled after amphetamine treatment did not differ between knockout and wildtype (Fig 2B). Testing mice in the light phase, GIT1 KO mice showed a drug-induced increase in locomotion at 4 mg/kg amphetamine similar to that of wildtype mice (Fig 2C and 2D). Testing at a more typical dose of 2 mg/kg amphetamine promoted increased locomotor activity in wildtype mice, but GIT1 KO mice failed to respond (Fig 2E and 2F). In this test, we also included GIT1-heterozygote mice, which appeared to have an intermediate response, but were not statistically significantly different rom either wildtype or KO. However, in neither the dark nor light phase tests did amphetamine suppress locomotion of GIT1 knockout mice to a level below that of wildtype mice.

**Fig 3:**
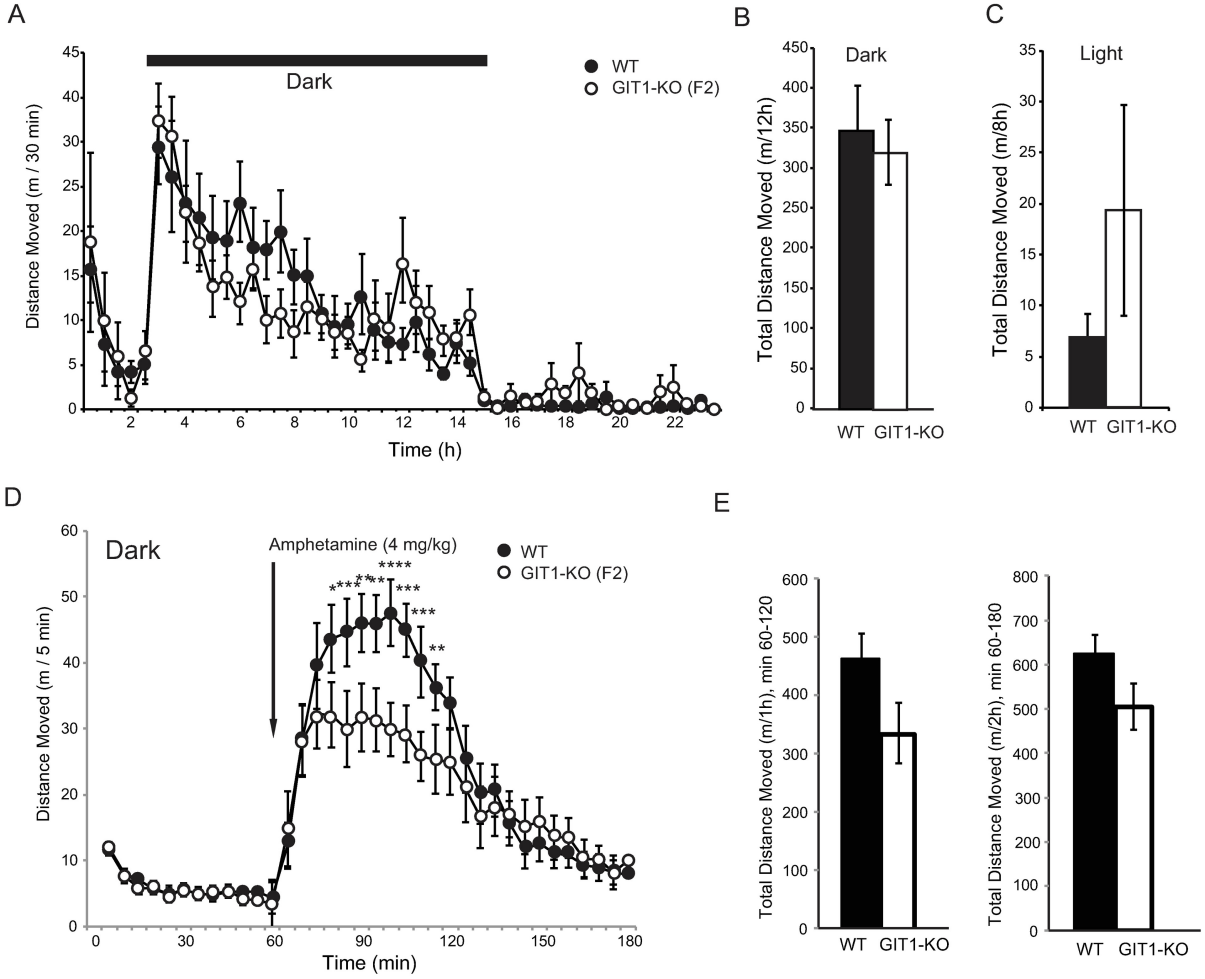
Spontaneous and amphetamine-induced locomotor activity in GIT1 deficient mice on the F2 c57Bl/6J x 129Ola genetic background. A) GIT1 WT (n = 8, black circle) and GIT1-KO (n = 8, open square) mice on the c57 x Ola F2 genetic background were subjected to the open field test for 24 hours under the normal 12hr-12hr light-dark cycle. High overall activity at time points during the post-dark light phase are due to distinct high-activity outlier individuals at each instance. Total distance was summed for the dark (active) phase (B) and light (inactive) phase (C) following the dark phase. D) GIT1 WT (n = 8) and GIT1-KO (n = 8) mice were injected with 4 mg/kg amphetamine in their active (dark) phase. E) Total distance was summed for 1h and 2h following the drug injection. *p*<0.001 in a two-way repeated measures ANOVA between genotypes over time; * *p*<0.05, ** *p*<0.01, *** *p*<0.001 within time using a Holm-Sidak post-hoc test.

GIT1-KO bred onto a c57Bl/6J background for 10 generations had negligible postnatal survival, precluding testing of living adult mice on this genetic background. To better match the genetic background used by Won and colleagues [19], we crossed c57Bl/6J-backcrossed *Git1* heterozygotes with 129P2/OlaHsd mice, and interbred the resulting F1 heterozygotes to generate F2 c57 x Ola knockout and littermate wildtype mice for testing. Compared to wildtype littermates, these c57 x Ola F2 mice also failed to demonstrate basal hyperactivity in 24 hour locomotor testing (Fig 3A), with both genotypes expressing equivalent activity in either the dark (Fig 3B) or the light (Fig 3C). While *Git1* F2 knockout mice did exhibit reduced locomotor activity at peak times after drug administration, they failed to show amphetamine-induced locomotor suppression (activity reduced below wildtype level) when tested in the dark phase of the diurnal cycle (Fig 3D) and did not differ from wildtype controls in total drug-induced locomotor distance (Fig 3E).

Thus, in our hands, neither critical aspect of ADHD-like behavior, namely basal hyperactivity or psychostimulant-induced hyperactivity suppression, is apparent in our strain of GIT1-deficient mice. Instead, GIT1 KO mice exhibited locomotor activation by the psychostimulant amphetamine, although abnormally so, with the GIT1-deficient mice appearing less sensitive to amphetamine compared to wildtype mice and showing a reduced response at some times after drug administration.

### Motor function is impaired in GIT1 mice

**Fig 4:**
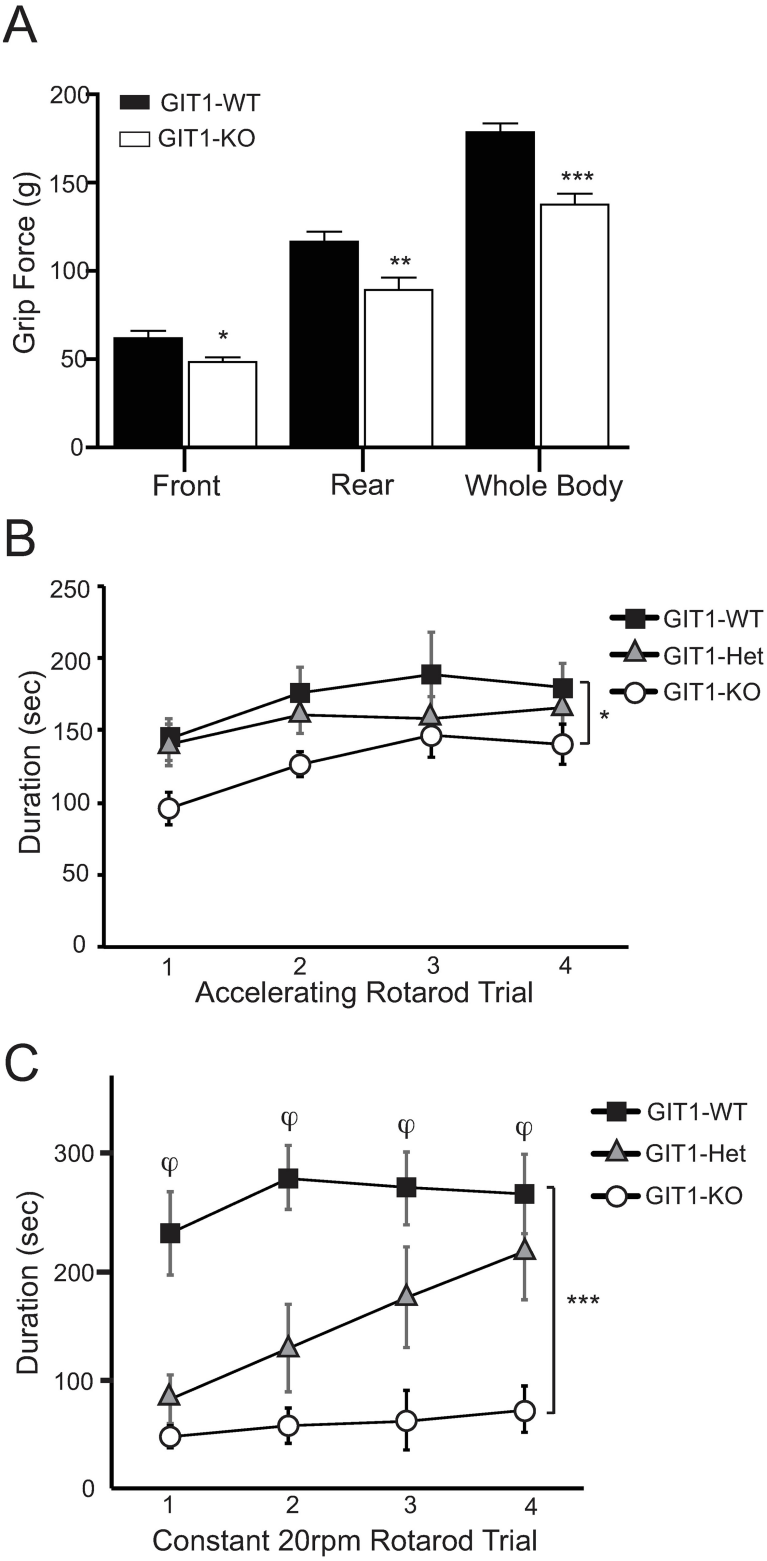
Sensory motor function and coordination in GIT1 deficient mice. A) Grip strength was assessed in GIT1 WT (n = 9, black bars) and GIT1 KO (n = 9, white bars) mice (* p = 0.0143; ** p = 0.0078; *** p<0.0001 in a two-tailed *t* test). B) Performance in the accelerated rotarod task was assessed in GIT1 WT (n = 9), GIT1 Heterozygote (Het, n = 8), and GIT1 KO (n = 9) mice (* p = 0.022 WT vs KO in a two-way repeated measures ANOVA; Het vs WT or KO was not significant). C) GIT1 WT (n = 9), GIT1 Het (n = 8), and GIT1 KO (n = 9) mice were challenged 24-hrs after the accelerated rotarod task with a 20rpm steady rotation of the rod (*** p<0.001 WT vs KO in a two-way repeated measures ANOVA; φ p<0.001 WT vs KO within trials using a Holm-Sidak post-hoc test).

The trend toward lower spontaneous activity and the reduced sensitivity to amphetamine-induced locomotor activity of GIT1-deficient mice could result in part from sensorimotor impairment. We therefore assessed sensory motor function of these mice by measuring grip strength and rotarod performance. Compared to wildtype littermates, GIT1 KO mice on a mixed genetic background demonstrated significantly less front-paw, hind-paw or four-paw grip strength (Fig 4A). Additionally, when challenged with the accelerated rotarod task, where speed of the rotating rod increases over time within a trial, the duration that GIT1 KO mice were able to remain running on the rod was significantly shorter than that of wildtype littermates across all trials, even as performance improved (Fig 4B). This sensory motor function deficiency was even more pronounced when these mice were challenged 24 hours later with a 20 rpm steady rotation of the rod, where GIT1 KO mice were markedly deficient at remaining on the rod (Fig 4C). GIT1 heterozygote mice appeared intermediate between KO and wildtype mice, but did not differ significantly from either in the accelerated rotarod task, and showed trial-dependent improvement in the 20rpm rotarod task. Taken together, these results provide evidence to suggest that GIT1 KO mice have motor function deficiency that may contribute to the alterations in spontaneous and psychostimulant-evoked locomotion.

### GIT1 KO mice show poor memory performance

Three distinct strains of GIT1-deficient mice have been reported by three groups to exhibit mild to severe learning and memory deficits in distinct tests: auditory fear conditioning [20], operant conditioning [18], and the novel object discrimination test and water maze [19]. We therefore tested our GIT1 KO mice on a mixed genetic background for learning and memory abilities in detail.

**Fig 5:**
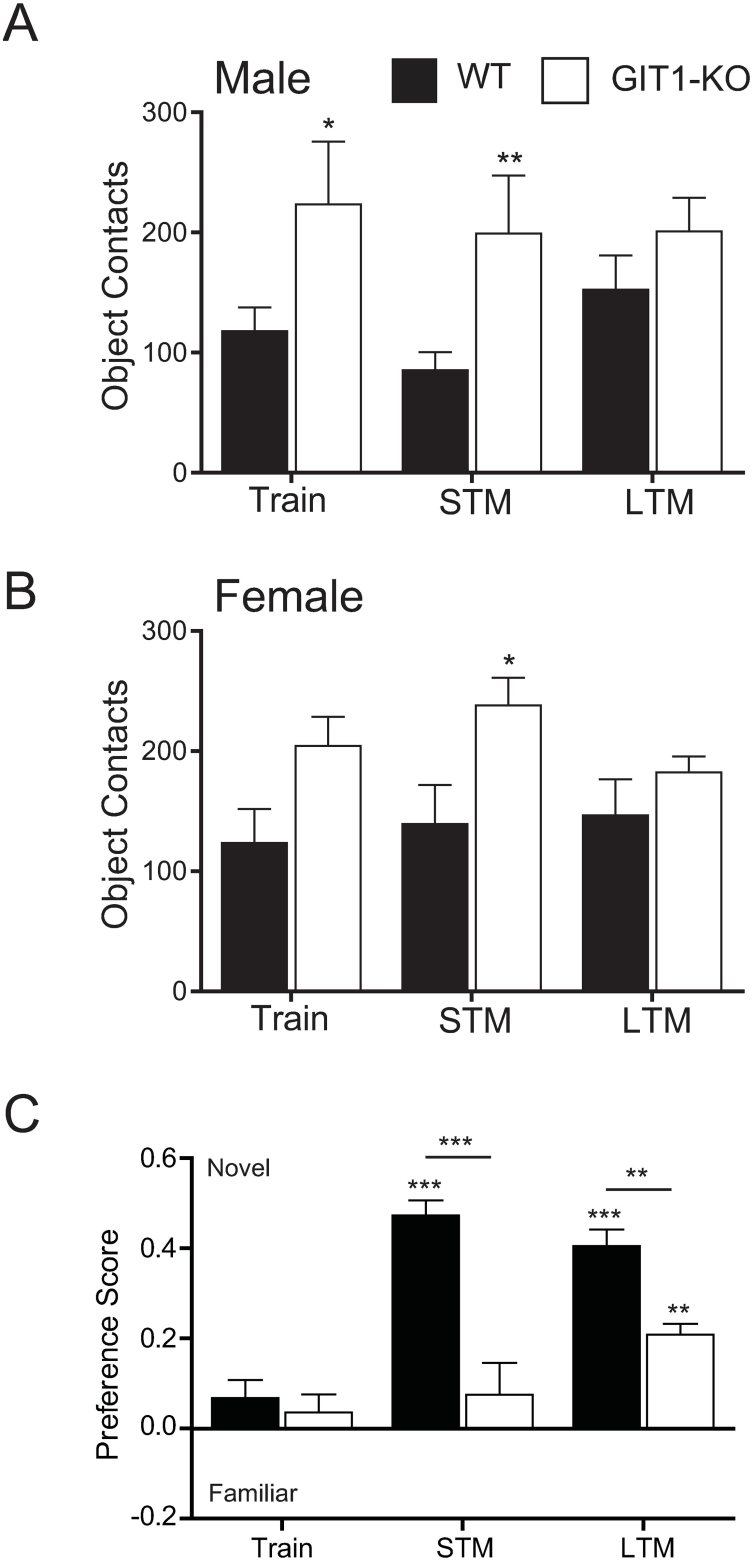
Short- and long-term novel object recognition is impaired in GIT1 KO mice. A,B) WT (n = 10, black bars) mice and GIT1 KO (n = 9, white bars) mice were exposed to test objects, and time spent in contact with the novel object (or during the training trial, the one that is replaced with the novel object) is shown for male (A) and female (B) mice. C) GIT1-KO mice demonstrated reduced preference for the novel object after 20 min (short-term memory, STM) and 24-hour (long-term memory, LTM) rest periods (*p*<0.001 between genotypes over trials in a two-way repeated measures ANOVA). Although by 24-hours, GIT1 KO mice demonstrated some preference for the novel object, performance in this task was poor compared to wild-type. *p<0.05, **p<0.01, ***p<0.001 within trial or between genotypes using a Holm-Sidak post-hoc test.

In the novel object recognition test, mice are tested for their ability to discriminate between a previously encountered object and a novel object. Compared to WT controls, both male and female GIT1 KO mice spent as much or more time in contact with test objects during all test periods, indicating that they had ample opportunity to learn to discriminate among objects (Fig 5A and 5B). Nevertheless, GIT1-KO mice exhibited impaired preference for exploration of a novel object when tested either 20 min (short-term memory, STM) or 24 hours (long-term memory, LTM) after the initial training encounter (Fig 5C). Although GIT1 KO mice did spend significantly more time with the novel object in the LTM test than in the STM test, this remained significantly less than wildtype controls. This suggests that GIT1-deficient mice exhibit defects in both short-term memory and long-term memory of previously encountered objects.

**Fig 6:**
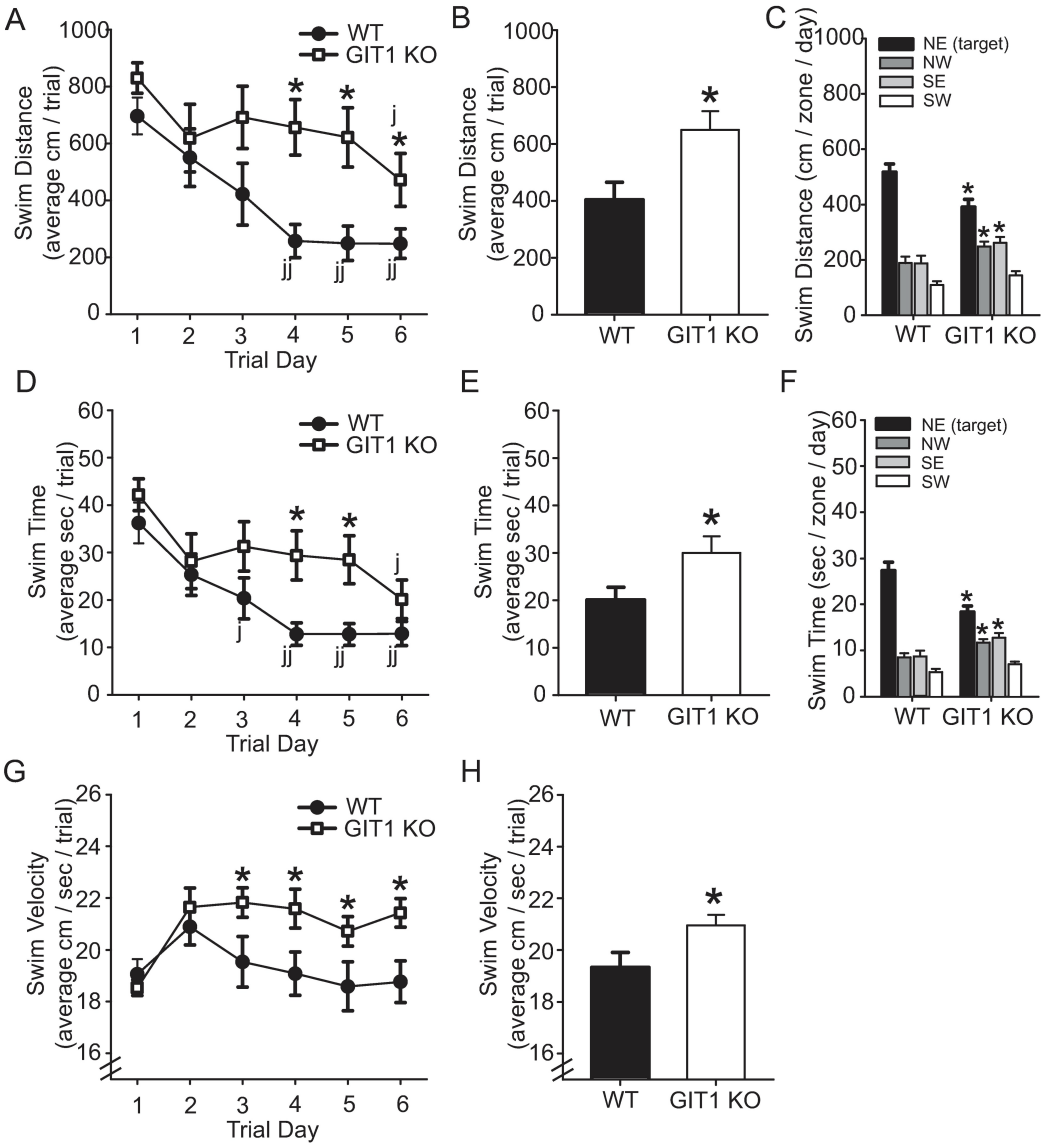
GIT1 KO mice show impairment in spatial memory in the Morris water maze. WT (n = 9, black symbols and bars) mice and GIT1 KO (n = 10, white symbols and bars) were trained over 6 days to find the hidden platform. Distance to find the platform (A and B), latency to find the platform (swim time, D and E), and swim speed (G and H) are shown. Swim distance and time were also analyzed for individual quadrants of the test pool (C and F). The average of two trials per day is plotted. All data is ± SEM. * *p*<0.05; ** *p*<0.01 in a two-way repeated measures ANOVA comparing KO to WT, followed by Holm-Sidak post-hoc within-trial comparison. φ *p*<0.05; φφ *p*<0.005 comparing daily performance of WT or KO to their own Day 1 performance. GIT1 KO mice traveled more distance and spent more time moving at a faster speed to find the hidden platform. GIT1 KO mice swam significantly less time and distance in the target quadrant, while swimming more time and distance in adjacent quadrants seeking escape.

The Morris water maze is a demanding learning paradigm that challenges spatial memory [28], in an arguably fear-driven environment. In the hidden platform (spatial) version of the test, wildtype mice showed a rapid decrease in swim distance (Fig 6A) to the platform by learning the position of the platform by day 4 of the 6-day training period, whereas GIT1 KO mice showed significant improvement in the task only by day 6 (Fig 6A and 6B). Closer examination of swim distance traveled revealed that while wildtype controls developed a direct swim path to the platform by day 4 of training by traveling more distance in the target quadrant compared to adjacent quadrants, GIT1 KO mice traveled less distance in the target quadrant and more distance in adjacent non-target quadrants (Fig 6C). Not surprisingly, the time taken to find the platform also was significantly longer for GIT1-deficient mice compared with wildtype controls (Fig 6D and 6E), and by day 4, GIT1 KO mice spent less time in the target quadrant and more time in adjacent non-target quadrants (Fig 6F). Due to this very poor learning performance, reversal training, where the platform is moved to a new location after the initial training is completed, was not tested. Because it is possible that poor performance by GIT1 KO mice in the physically challenging Morris water maze task could potentially be explained in part by their neuromuscular coordination deficiency (see Fig 4), we therefore assessed swimming speed as a measure of coordination performance. However, GIT1 KO mice were not impaired in this parameter, and by the third day of training, swim speed of GIT1 KO mice was significantly faster than that of WT controls (Fig 6G and 6H). This may partially be due to the fact that GIT1 KO mice spend more time active in the pool, and are therefore able to increase their swim velocity over time, compared to wildtype mice that have learned the position of the platform by day 3 and therefore spend minimal time swimming in the pool.

**Fig 7:**
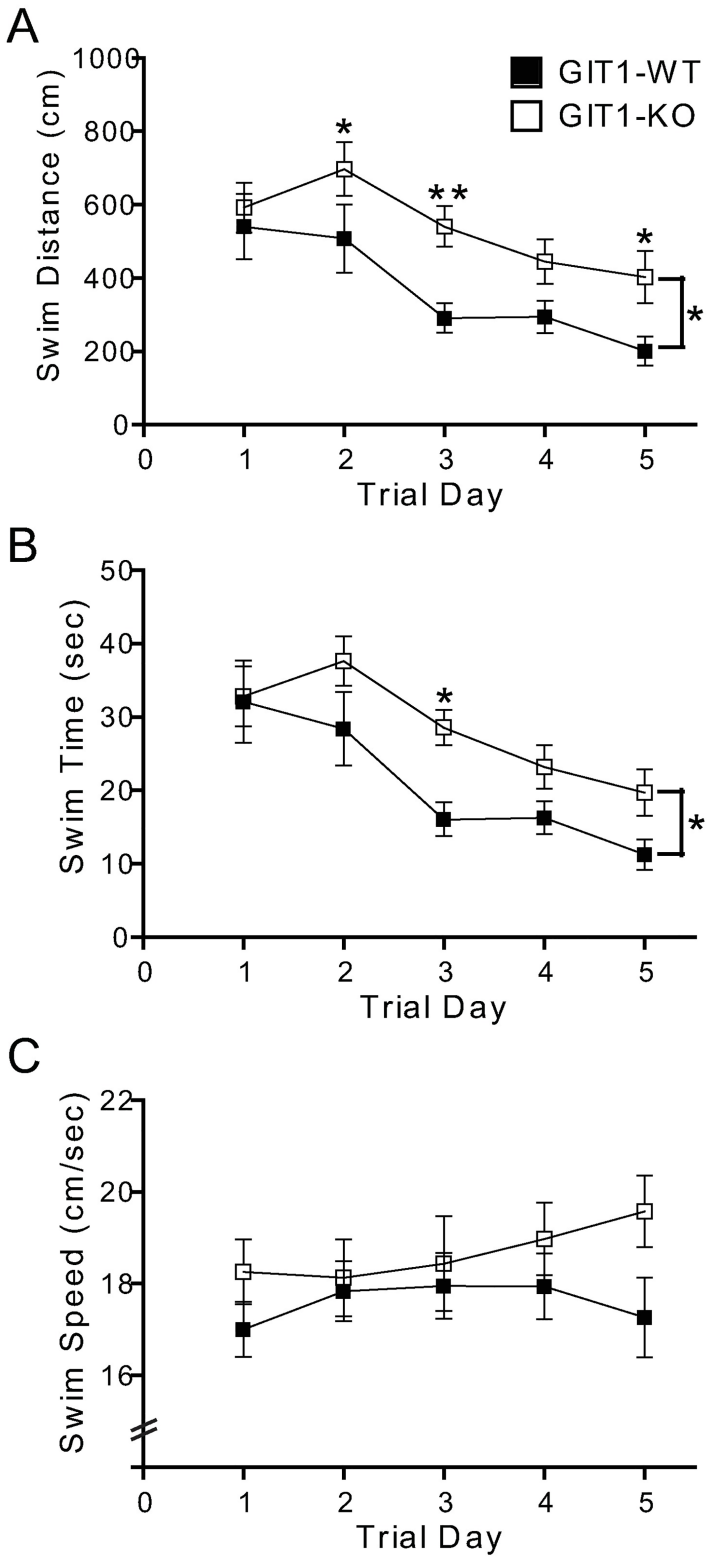
GIT1 KO mice show impairment in the visible platform version of the Morris water maze. GIT1 KO mice (open square, n = 9) and WT (closed circle, n = 9) were subjected to the visible platform version of the Morris water maze. Distance to find the platform (A), latency to find the platform (swim time, B), and swim speed (C) are presented. The average of two trials per day is plotted. All data is ± SEM. * *p*<0.05; ** *p*<0.01 in a two-way repeated measures ANOVA, followed by Holm-Sidak post-hoc within-trial comparison.

The precise role of GIT1 in cognition is unknown, but with its widespread expression in the brain [13], it is possible that GIT1 could regulate cognitive processes that drive motivation to escape to account for the deficiencies observed here, as well as those reported by others [18–20]. To assess the role of GIT1 in escape motivation, the visible platform version of the Morris water maze task was used [29]. Similar to the hidden platform version, wildtype mice demonstrated rapid improvement in swimming to the visible platform over the five days of testing (Fig 7). GIT1 KO mice also showed improvement in this task across test days, although they traveled significantly longer distances to reach the platform (Fig 7A) and were significantly slower in arriving at the platform (Fig 7B), despite a higher swim velocity than wildtype littermates (Fig 7C).

Taken together, these data provide evidence to suggest that motivation to escape may be mildly impaired in the absence of GIT1, which may contribute in part to the cognitive deficiency in GIT1-deficient mice. However, the improvement seen by GIT1 KO mice in the visible platform Morris water maze indicates that the extent of this defect is likely insufficient by itself to explain the severe spatial memory impairment in the hidden platform spatial memory version of the Morris water maze task. We conclude that GIT1 indeed plays a direct and key role in spatial memory cognition.

### Synaptic plasticity is impaired in hippocampal neurons of GIT1 KO mice

Synaptic activation of Cdc42 leads to localized activation of PAK kinases, which in turn regulate cytoskeletal rearrangements responsible for various aspects of synaptic plasticity [25, 30]. The PIX protein partners of GIT1 are Cdc42/Rac1 guanine nucleotide exchange factors as well as scaffolds for PAK [2], suggesting that loss of GIT1 will lead to mislocalization of PIX/PAK to disrupt Cdc42/Rac1- and PAK-dependent functions within neurons. Further, loss of GIT1 has been reported to lead to substantial reduction of PIX proteins in the brain [19], and we confirm this reduction in PIX levels, but not of PAKs, in brain of our GIT1-KO strain (Toth et al, submitted). Thus GIT1 deficiency not only leads to loss of GIT1-dependent mechanisms for PIX/PAK localization, but also to loss of PIX that might localize PAK through binding to other partners. Together the loss of GIT1 and most PIX is expected to severely disrupt Cdc42/Rac1- and PAK-dependent functions in neurons.

**Fig 8:**
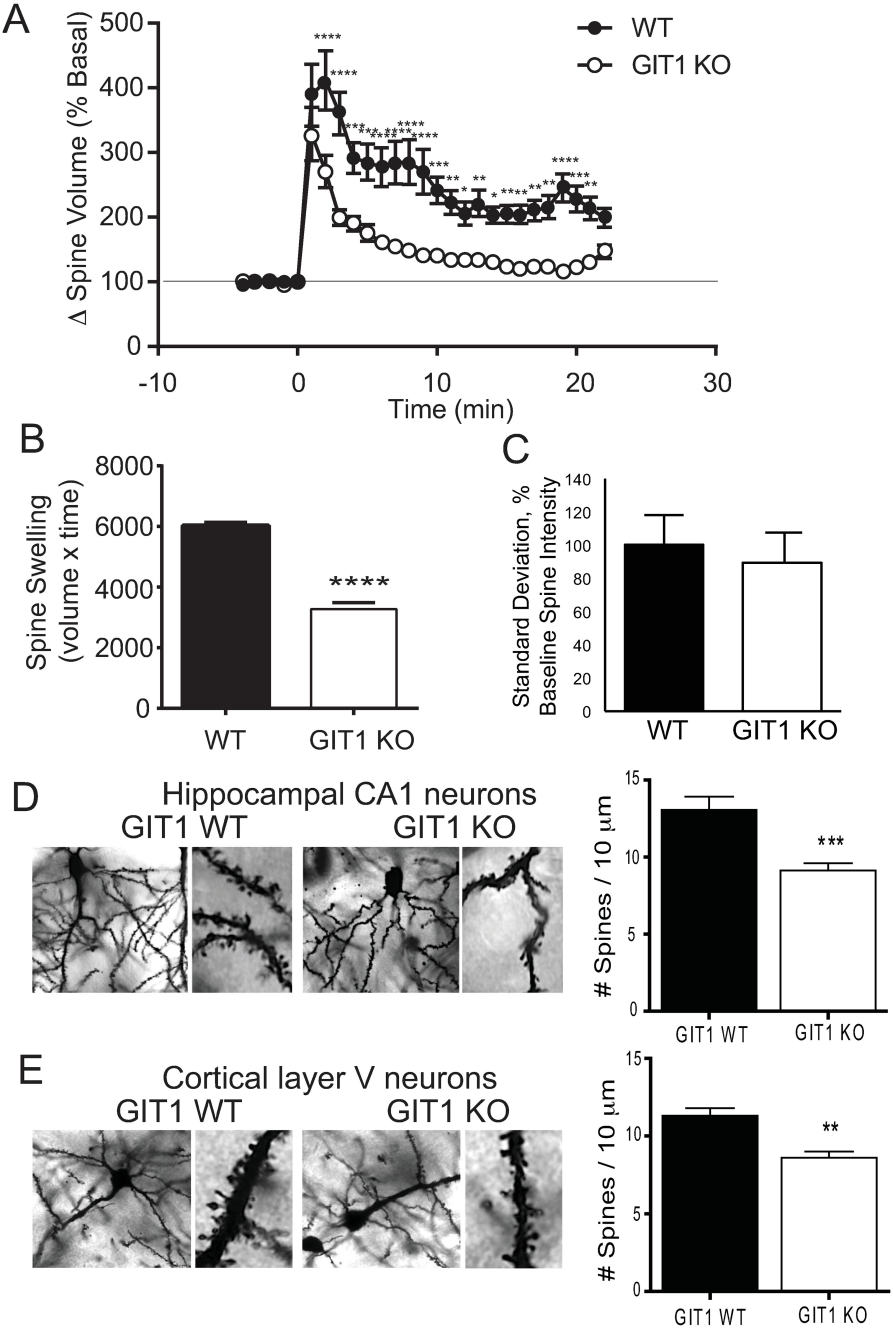
Structural plasticity of single dendritic spines from hippocampal CA1 neurons. Brain slices from GIT1^flox/flox^ mice transfected with GFP alone or GFP with tdTomato-Cre recombinase were cultured for 9–11 days post-transfection, and individual spines were visualized surrounding 2-photon glutamate uncaging (A). Spine volume change is plotted as fold change from basal size. * *p*<0.05; ** *p*<0.01; *** *p*<0.005, **** *p*<0.001 in a two-way repeated measures ANOVA, followed by Holm-Sidak post-hoc within-trial comparison. Area under the curve (volume x time) was summed to indicate the scale of spine swelling over the test period (B). **** *p*<0.001 in a 2-tailed *t* test. Dendritic spine density in GIT1-KO hippocampal CA1 neurons (C) and in cortical layer V neurons (D). Brain slices from GIT1 WT (n = 10) and GIT1 KO (n = 10) were Golgi stained and imaged using confocal microscopy. Projecting spines on well-resolved neural processes over 10 μm that emanated from the soma were counted, and divided by length of process for each neuron. ** *p* = 0.01, *** *p* = 0.005 in the two-tailed *t* test.

One such function is synaptic structural plasticity, whereby strongly activated synaptic spines undergo cytoskeletal rearrangements that result in an enlargement of the spine head, and a concomitant increase in synaptic neurotransmitter sensitivity underlying long-term potentiation [31]. We therefore examined structural plasticity in hippocampal spines lacking GIT1. Brain slices from *Git1*^flox/flox^ mice were transfected with GFP plus Cre recombinase fused to tdTomato to induce inactivation of the GIT1 gene in Cre-transfected neurons, or with GFP alone as control. Single hippocampal CA1 neuron spines were stimulated using 2-photon glutamate uncaging, and visualized by 2-photon microscopy. Consistent with previous reports [25, 30, 32, 33], glutamate uncaging at control spines induced a rapid increase in spine volume lasting for 1–2 minutes, that was followed by a lower but sustained spine volume increase lasting for >20 minutes (Fig 8A). Conversely, spines from cells expressing Cre recombinase displayed a blunted spine volume increase that was not sustained, indicating impaired structural plasticity in dendritic spines lacking GIT1. Summing volume measurements over time demonstrates a significant overall reduction in spine volume expansion in activated spines in the absence of GIT1 (Fig 8B). Importantly, the magnitude of the volume increase of dendritic spines during structural plasticity has been shown to be highly correlated with the change in AMPA receptor-mediated currents associated with the expression of LTP [33], suggesting that the impaired structural plasticity observed in the absence of GIT1 is likely commensurate with a reduction in the functional potentiation of these synapses. To control for spine size-associated variation, we also assessed the variability of baseline spine size dynamics in the period prior to glutamate release (Fig 8C), and found this to not differ between wildtype and KO. It has been reported previously in a distinct GIT1-knockout strain that hippocampal CA1 neurons have reduced spine density [18]. To quantify synaptic spine density in our strain, brain sections from GIT1 KO and WT mice were Golgi stained, and hippocampal neuron spines were counted (Fig 8D). Consistent with a prior report, our GIT1 KO mice have reduced synaptic spine density in hippocampal CA1 neurons. Similarly, GIT1-KO mice also show significantly reduced spine density in cortical layer V pyramidal neurons (Fig 8E), a neuronal type similar to hippocampal CA1 pyramidal cells. Taken together, these data provide evidence that GIT1 functions within postsynaptic spines to help regulate proper cytoskeletal rearrangements underlying structural plasticity after synaptic activation, consistent with a role in synaptic plasticity during learning, as well as acting to regulate spine number.

## Discussion

Here we report detailed analysis on motivational learning and memory in the absence of GIT1 expression in the mouse. GIT1 KO mice display severe deficits in hippocampal-driven learning and memory, as observed in the Morris water maze and novel object recognition task. We confirmed that loss of GIT1 leads to reduced dendritic spine density in hippocampal CA1 neurons, as well as in cortical neurons. Loss of GIT1 additionally impairs the activity-dependent, cytoskeleton-driven structural plasticity of single dendritic spines, suggesting a role for GIT1 in the expression of synaptic plasticity.

The locomotor data presented here is consistent with our initial characterization of the GIT1 KO line, but contradicts a highly touted [34, 35] report suggesting the involvement of GIT1 in ADHD-like hyperactive behavior [19]. We previously showed that older (8 month old) adult GIT1-KO mice had relatively normal locomotor activity in the open field during a screen for anxiety-like behavior. Using a distinct genetrap line disrupting GIT1 expression, Won *et al*. described basal hyperactivity during the active night phase, but not during the day, in young adult mice (2 months) but not older adult mice (8 months) [22]. Here, we examined diurnal basal activity as well as novelty- and psychostimulant-induced locomotor behaviors, in young GIT1-KO mice. Instead of hyperactivity, we find normal to slightly hypoactive activity during both the night and day. Activity of GIT1-KO mice is elevated, not reduced, by acute doses of the psychostimulant amphetamine, although GIT1-KO mice exhibit reduced sensitivity to amphetamine compared to wildtype. Thus, in our tests, we find no evidence supporting the contention that loss of GIT1 induces an ADHD-like state in mice, as we fail to see either of the two hallmarks of ADHD: basal hyperactivity, or paradoxical psychostimulant-induced locomotor calming. It was suggested that the loss of mouse hyperactivity with age might be similar to the loss of hyperactivity often observed in ADHD patients as they progress beyond adolescence [19], but this is clearly not the case in our strain if GIT1-KO mice.

There are several potential explanations for this discrepancy between these studies. First, the mice studied bear distinct alleles: our knockout allele is a markerless deletion of exons 2–7 that would have out-of-frame splicing of exon 1 to exon 8, while the strain used by Won and colleagues bears a genetrap that inserts a *Lac*Z exon cassette and hygromycin resistance gene into the first intron of the *Git1* gene [22]. While both strategies result in the apparent complete loss of GIT1 protein, the inserted *Lac*Z and hygromycin genes may have effects not due to loss of GIT1 such as affecting neighboring genes, or less likely, the genetrap mice may harbor an additional genetrap insertion in another gene that modifies locomotor activity. Second, the two strains are on different genetic backgrounds, which may affect phenotypic expression. Won et al used a defined F2 c57 x 129 background, while our mice are a mixed c57 x 129 background for most tests. Like Won and colleagues, we have found knockout pup survival in the backcrossed c57BL/6J background was too poor to obtain sufficient adult animals for study, but we have also tested locomotor activity in GIT1 KO mice bred to a defined c57BL/6J x 129Ola F2 background, and also seen no hyperactivity. Third, there are discrepancies between the locomotor data presented by Won and colleagues and data with other hyperactive lines reported in the literature (e.g., [36, 37]. The reported level of locomotor activity of their GIT1 littermate wildtype control mice is very high at 30 meters in the first 10 min in the test chamber, 10 times higher than seen with other wildtype strains [36]; this is roughly equivalent to the hyperactivity observed in mice deficient in the dopamine transporter (DAT), an extremely hyperactive line used as a model for some aspects of ADHD [37]. These wildtype mice also show little habituation to the novel test chamber over the 50 min test period, suggesting that other factors might be influencing the expression of locomotor behavior in these mice under those test conditions. Whatever the explanation, it is clear that loss of GIT1 by itself is insufficient to produce robust ADHD-like behavior. This may explain the later report of a lack of association of *GIT1* with ADHD in a study of Brazilian children [38] and a recent report failing to find an association of *GIT1* with ADHD in three large cohorts [39]. At this point we can only speculate why results differ so dramatically between our respective studies, but elements beyond loss of GIT1 function per se must be important in expressing the ADHD-like behavior observed by Won and colleagues [19]. A first step in resolving the differences would be to test these two GIT1 deficient lines in the different environments and procedures. Furthermore, two additional GIT1-deficient mouse lines exist that could be tested for locomotor/ADHD-like phenotypes: a traditional NEO cassette-exon replacement knockout [18] and an independent derivation of mice bearing the *GIT1* genetrap allele [40].

In contrast to the discordant observations of locomotor activity, three distinct lines of GIT1-deficient mice have shown moderate to severe learning and memory deficits [18–20]. Because the GIT1 partner α-PIX and its partner PAK3 are known human X-linked intellectual disability genes [14, 15], it was expected that GIT1 might be important for learning and memory [8, 16]. Previous reports have implicated GIT1 in the regulation of dendritic spine morphogenesis and synapse formation in primary hippocampal neurons [8, 16]. We have reported that GIT1-KO mice exhibit poor learning in a classical aversive learning paradigm, auditory fear conditioning [20]. The Berk group reported that their traditional NEO replacement GIT1-KO strain showed poor learning in an operant conditioning paradigm using a rewarding sweet treat [18], and the Kim group demonstrated poor spatial learning in the Morris water maze and poor novel object recognition in their GIT1 genetrap strain [19]. This agreement across multiple strains and tests is consistent with a robust learning deficiency phenotype. Furthermore, *Drosophila* lacking the sole GIT protein, dGIT, also demonstrated dendritic spine, synapse and synaptic vesicle recycling defects [11, 39]. Here we show that our GIT1-KO strain has learning deficits in the Morris water maze and in novel object recognition as well. GIT1-deficient mice appear to be a robust model for human disorders involving learning and memory deficits. Further experiments are required to establish what human conditions GIT1 deficiency models most closely and whether the reported promoter-like activity of the ADHD-associated single nucleotide polymorphism in the human *GIT1* gene [19] is functionally relevant *in vivo*.

Accompanying the learning impairment in our GIT1 KO mice was a reduction in spine density in hippocampal and cortical neurons, and a deficit in the maintenance of activity-dependent spine enlargement in hippocampal neurons. Mistargeting of GIT1 to prevent its localization within synaptic spines in cultured rat hippocampal neurons has been reported to reduce spine density [8], and GIT1 knockdown in these neurons also reduces spine density [16]. A distinct GIT1 KO strain also has been reported to have reduced spine density [18]. Reduced spine density is a common feature of X-linked intellectual disability and other learning defects [41]. The molecular mechanisms related to the deficit in structural plasticity in GIT1-deficient mice are not understood, but are likely to be related to alterations in GIT/PIX signaling cascades that contribute to the postsynaptic induction of NMDA receptor-dependent synaptic plasticity through partners such as liprin-α [10] or Ephrin-Grb4 [42], as well as presynaptic neurotransmitter vesicle release and recycling defects [11, 43]. The similarity of the GIT1 KO phenotype to the PAK1/PAK3 double-knockout phenotype [44] and α-PIX-KO phenotype [45] suggests that loss of localized PAK activation mediated by synaptic GIT/PIX complexes may be a primary defect. The lack of GIT1 leads to immediate loss of GIT-dependent localization of PIX/PAK within neurons, but long-term lack of GIT1 also destabilizes PIX proteins, further reducing PIX functions that are independent of GIT1/PIX complex stabilization. A report of a role for GIT1 regulation of Arf1-Pick1 during synaptic plasticity [46] suggests that loss of GIT1 ArfGAP activity within synapses may also contribute to the observed learning deficit. At the molecular level, learning requires synaptic plasticity that employs glutamatergic neurotransmission to promote cytoskeletal rearrangements for altering synaptic strength [31], and GIT1 has been reported to play a role in the pathway from glutamate receptors to cytoskeletal rearrangements [9]. Hence, we conclude that GIT1/PIX signaling in the hippocampus is important for synaptic plasticity, object recognition and spatial memory consolidation. Future experiments are needed to dissect the precise mechanisms involved in these processes.

Additionally, we show that GIT1 KO mice have a motor function deficiency, which may complicate the behavioral outcome in many tests that require motivation to move and explore. Motor function is slightly impaired in these mice as measured using rotarod, and the visible platform Morris water maze revealed a slight decrease in the motivational drive to swim to the platform, both which may contribute to the observed decrease in locomotion. However, increased swim speed in the Morris water maze highlights that these animals are able to perform coordinated locomotion when necessary. In contrast, the ability of GIT1-KO mice to interact with identical objects equivalently during training suggests that visual sensory function is not confounding results in object recognition, or in the water maze. It should be pointed out that behavioral and cognitive outcomes show a high degree of complexity involving a multitude of molecular signaling events. Our data suggest that motor function may be a contributing factor to the decreases we observed in locomotor activity in the GIT1 KO mice, and the deficit in spatial memory observed in GIT1-deficient mice is due to altered synaptic plasticity, but we cannot exclude the possibility that decreased GIT/PIX signaling also interfere with motivational processes that affect the behavioral outcome of cognitive testing.

In summary, by using our GIT1 KO mouse strain, we determined that reduction of GIT/PIX signaling alters synaptic plasticity in the hippocampus, resulting in deficits in short-term and long-term spatial memory and recognition memory. We also report a novel role for GIT1 in motor function and in motivation, contributing to decreases in spontaneous locomotion. However, we find no evidence to support an ADHD-like phenotype due to the absence of GIT1 in a markerless knockout allele, suggesting that this reported phenotype is somehow specific to the strain of GIT1 KO used in that study [19]. We have also recently characterized learning-related behavior in mice lacking GIT2, and also find no evidence for an ADHD-like phenotype but also find that GIT2 does not regulate learning and memory function due to brain GIT2 being a splice variant that does not form GIT2/PIX complexes (Toth *et al*., submitted). By defining the specific roles of GIT/PIX signaling in learning and memory through targeting of GIT1 versus GIT2, our experiments provide novel insights on signaling and scaffolding functions that may be used as potential targets to compensate for learning and memory deficits.
